# A Cross-Disciplinary Analysis of the Complexities of Scaling Up eHealth Innovation

**DOI:** 10.2196/58007

**Published:** 2024-12-02

**Authors:** Sanne Allers, Chiara Carboni, Frank Eijkenaar, Rik Wehrens

**Affiliations:** 1 Erasmus School of Health Policy & Management Erasmus University Rotterdam Rotterdam Netherlands

**Keywords:** innovation, eHealth, remote patient monitoring, scale-up, cross-disciplinary, qualitative case study, health care systems, adaptation, complexity, health care, framework, ecological perspective, barriers and facilitators

## Abstract

Innovative eHealth technologies are becoming increasingly common worldwide, with researchers and policy makers advocating their scale-up within and across health care systems. However, examples of successful scale-up remain extremely rare. Although this issue is widely acknowledged, there is still only a limited understanding of why scaling up eHealth technologies is so challenging. This article aims to contribute to a better understanding of the complexities innovators encounter when attempting to scale up eHealth technologies and their strategies for addressing these complexities. We draw on different theoretical perspectives as well as the findings of an interview-based case study of a prominent remote patient monitoring (RPM) innovation in the Netherlands. Specifically, we create a cross-disciplinary theoretical framework bringing together 3 perspectives on scale-up: a structural perspective (focusing on structural barriers and facilitators), an ecological perspective (focusing on local complexities), and a critical perspective (focusing on mutual adaptation between innovation and setting). We then mobilize these perspectives to analyze how various stakeholders (n=14) experienced efforts to scale up RPM technology. We provide 2 key insights: (1) the complexities and strategies associated with local eHealth scale-up are disconnected from those that actors encounter at a broader level scale-up, and this translates into a simultaneous need for stability and malleability, which catches stakeholders in an impasse, and (2) pre-existing circumstances and associated path dependencies shape the complexities of the local context and facilitate or constrain opportunities for the scale-up of eHealth innovation. The 3 theoretical perspectives used in this article, with their diverging assumptions about innovation scale-up, should be viewed as complementary and highlight different aspects of the complexities perceived as playing an important role. Using these perspectives, we conclude that the level at which scale-up is envisaged and the pre-existing local circumstances (2 factors whose importance is often neglected) contribute to an impasse in the scale-up of eHealth innovation at the broader level of scale.

## Introduction

Innovative eHealth technologies, defined in this paper as tools that support the organization and delivery of health services and information using the internet and related technologies [1], are becoming increasingly common in health care systems worldwide. These technologies have been discussed in the literature with careful enthusiasm, with studies often weighing challenges related to “privacy, liability, and costs” [2] against their potential to support patient-centered care, remote patient monitoring (RPM), and prevention [3-6]. In addition, eHealth technologies have become increasingly central in policy debates, with policy makers consistently articulating high expectations around eHealth’s role in health care’s future sustainability.

The literature often singles out the fragmentation of the eHealth landscape as a potential hindrance [7,8]. Such fragmentation is found to result in various problems, including nondissemination of valuable innovations [9]; inequalities resulting from a failure to reach patients who have the greatest needs [10] or who reside in specific areas [11]; and generally unsustainable implementation, given that eHealth providers need to achieve a considerable level of coverage to become (economically) viable [12].

Scale-up is often proposed as a remedy in this context [13]. For instance, policy makers have advocated replicating proven eHealth technologies within and across health care systems. In its global strategy 2020-2025, the World Health Organization (WHO) recommends focusing on nationwide scaling of eHealth technologies, proposing that principles such as *scalability* and *replicability* should be at the heart of current efforts around eHealth development and implementation [14]. Many national governments have also attempted to support the scale-up of eHealth technologies, emphasizing that stakeholders should share and adopt best practices rather than reinvent the wheel [15,16].

Despite this dominant rhetoric, examples of successful eHealth technology scale-up are few [17,18]. Widespread adoption is plagued by the “diffusion chasm,” with a gap opening up between initial invention and successful market penetration [19]. A recent literature review, for example, found that none of the eHealth technologies implemented in the United Kingdom managed to reach organization-wide or large-scale adoption [20]. Similarly, a publicly funded program in the Netherlands aimed at introducing eHealth technologies nationwide [21] ultimately fell short of its goal, that is, to counteract fragmentation and encourage further scale-up of local initiatives [22].

Although the poor scale-up of eHealth innovations is widely acknowledged [18], we still have only a limited understanding of why scaling up eHealth technologies is so challenging. So far, research has described the stagnation of eHealth implementation without articulating clear strategies to overcome it [23,24]. These studies focus on the local implementation of pilot eHealth technologies and assume that this process has a clear beginning (ie, the introduction of an innovation in a specific organizational setting) and an end (ie, the innovation being structurally embedded in that specific organizational setting). What came before and what comes after are often left out of the picture. Thus, although researchers acknowledge the necessity of scale-up for sustainable eHealth innovation, they typically end up studying implementation issues as though they were separate from scale-up [25]. This narrow approach does not contribute to our knowledge of what scaling up eHealth (and the complexities associated with it) entails or our knowledge of promising strategies to facilitate scale-up.

This article seeks to address this knowledge gap by answering the question of what complexities are encountered when attempting to scale up an eHealth technology and what strategies are applied by stakeholders to deal with these complexities. We start by describing and combining insights from 3 existing theoretical perspectives on technology scale-up in and beyond health care. We then bring these insights to bear on eHealth by examining a case study of a Dutch eHealth technology that, despite being considered a success at the local level, encounters major (and common) challenges when being scaled up. By mobilizing empirical insights stemming from the case analysis, our discussion contributes to existing theorizations of the complexities of scale-up, thus illuminating other dimensions of these difficulties as stakeholders experience them in practice.

## Theoretical Perspectives and Insights From a Case Study

### Meaning of Scale-Up

Numerous common definitions of scale-up in the literature fall into 2 categories. The first describes scale-up as *the replication* of an existing innovation in “multiple geographic locations and contexts to maximize the number of people that an innovation reaches” [26]. Conversely, the second describes scale-up as *the gradual adaptation* an existing innovation undergoes as it becomes embedded in more and more dimensions of health care practice (ie, covering more patients, involving more providers, or adding to the steps involved in care provision). Although they differ in how they regard the innovation itself, both definitions consider the core of scale-up to be an expansion of an innovation’s *coverage*. Consistent with this, Spicer et al [27] defined scale-up as “an increase in the coverage of health interventions that have been tested in pilot and experimental projects in order to benefit more people.” This paper defines scale-up, in the broadest sense, as the steps taken to progressively expand the coverage of an existing innovation.

### Theoretical Perspectives on Innovation Scale-Up

In this section, we offer a high-level discussion of a number of theoretical perspectives that various disciplines have developed to think through the complexities associated with innovation scale-up. The 3 cross-disciplinary perspectives we have selected are by no means an exhaustive list of the theoretical approaches to implementation and scale-up proposed in the literature. They do, however, mobilize a diversity of viewpoints and arguments that enable us to illuminate crucial aspects of the complexities of technology scale-up, giving us a generative heuristic framework for our analysis. Selected precisely by virtue of their differing approaches, the 3 perspectives synthesize a variety of middle-range theories stemming from conventional management scholarship, complex adaptive system theory, and critical social science approaches, respectively, but for the sake of brevity, we refer to them as structural, ecological, and critical perspectives. Although these labels might appear arbitrary, we argue that they emphasize crucial aspects of each perspective: a structural perspective’s focus on high-level mechanisms and a somewhat immobile social world; an ecological perspective’s emphasis on interactions of different (human) components of health systems; and a critical perspective’s attempt to politicize scale-up as well as subvert its commonsense conceptualizations.

In what follows, we first illustrate these perspectives’ conceptualizations of the complexities around and solutions for enabling scale-up. Subsequently, we combine these theoretical perspectives to analyze a real-world eHealth innovation case.

#### Structural Perspective

The structural perspective, particularly prominent in economic and management theories, foregrounds the role of structural system barriers in hindering scale-up beyond the local level, with the possibility of scale-up resting on the removal of these barriers. For instance, Wang et al [28], in their study of telehealth adoption in the United States, found that the medical-legal framework of health care delivery impedes the successful scale-up of telehealth. As they argued, “policymakers must rethink and address the economic incentives and payment of telehealth services, the medical-legal issues surrounding virtual care, and the effects of increased competition across geographic areas and jurisdictions.” Similarly, the scoping review by Gijsbers et al [29] concluded that “successful upscaling of telemonitoring requires insight into its critical success factors, especially at an overarching national level. […] A wide program on change management, nationally or regionally coordinated, is key.” In summary, the structural perspective proposes that system-wide innovation scale-up depends on overcoming all critical barriers, assuming that it is possible to adjust systems to remove such barriers.

#### Ecological Perspective

The ecological perspective, rooted in organization studies and health systems research, emphasizes the local interrelated factors that must be considered to scale an innovation locally and replicate it in another setting. The analysis by Greenhalgh and Papoutsi [30] of the different logics that challenge the dissemination of innovations exemplifies this perspective: “Complexity can be hard to square with spread strategies that seek to replicate a ‘blueprint’ innovation in a standardized way across widely different settings. The plan-do-study-act engine might work for small-scale improvement initiatives, but spreading and scaling up major innovations across a health system requires attention to the underlying logic of complex systems, which is ecological rather than mechanical.”

In another study, Greenhalgh et al [31] argued that explaining the complexities of innovation in terms of barriers and facilitators does not sufficiently acknowledge the intricacies of the setting in which it is introduced. Studies that we group under the ecological perspective emphasize, for example, the importance of conducting sensemaking work among stakeholders before replicating an innovation [32]. Others emphasize the need to acknowledge the dynamic relationship between different factors in a local setting [31], or argue that an innovation must fit in with the diverging institutional logics of all relevant stakeholders before they can accept it as part of their practice [33]. In short, the human and technical characteristics of the local setting (eg, belief systems of local providers, technical interoperability of adopted and pre-existing systems, and stakeholders’ clashing institutional logics) must be acknowledged and worked on before an innovation can be scaled from another context. An example of a widely used theory applying this perspective is the RE-AIM framework, which fits in with a social-ecological perspective [34]. It conceptualizes the impact of innovative programs as depending on the percentage and characteristics of the people who receive or are affected by the intervention. Eventually, its aim is for innovations “to become a relatively stable, enduring part of the behavioral repertoire of an individual, organization or community.” In other words, the ecological perspective postulates that a successful innovation scale-up requires work to adapt the local setting so that it will accommodate the innovation. 

#### Critical Perspective

A more critical perspective on scale-up has been formulated by authors active in the field of science and technology studies (STS). For instance, in their article on the politics of scaling, Pfotenhauer et al [35] described scaling as a current “obsession of innovation discourses and, with it, contemporary social, political and economic life at large.” Critical reflections on scaling often build on Anna Tsing’s definition of scalability as the ability of a system to “expand without changing” [36] and, according to Hanna and Park, without “rethinking its constitutive elements” [37]. These authors shed a different light on the local uniformity that the ecological perspective posits as necessary for scaling up: adapting local systems generates tensions because it disregards the diverse ways in which people define problems and solutions, priorities, and values. As Pfotenhauer et al [35] argued, any narrative presenting scaling up as a smooth process is suspicious because it probably excludes certain perspectives. Hanna and Park [37] argued that the very idea of scalability as replication entails that the work that sustains innovation needs to be something “interchangeable, abstract, and universal.” Thus, in scaling-up discourse, the emphasis ends up being on the standardization of infrastructure, for instance, at the expense of more relational views that stress the inherently more unpredictable and therefore flexible work needed to maintain networks of humans and technologies. In other words, the critical perspective describes the need to examine innovations in their specific context of emergence, assuming that during implementation, both the innovation and the local setting are reshaped in a work-intensive process of mutual adaptation, and that some type of local knowledge or practice is inevitably lost in this process. Unlike the ecological perspective, the critical perspective suggests that replicating a blueprint innovation in a new setting requires work to adapt not only the context but also the innovation itself.

#### Cross-Disciplinary Framework

Table 1 summarizes the characteristics of the 3 perspectives on innovation scale-up. We have combined the 3 perspectives into a cross-disciplinary framework, with each one making a unique contribution.

The structural perspective advocates the removal of systemic barriers and the strengthening of facilitators, assuming that the changes required to stimulate scale-up can be pinpointed. This perspective contributes to our framework by focusing on the structural facilitators and barriers (eg, regulatory and financial) that emerge in systems and that support or hinder innovation up-scaling.

The ecological perspective teaches us that conditions at the local level are complex and diverse, and we cannot expect to know in advance what needs to be done for an innovation to “work” in a specific setting. Local complexity must be considered before introducing an innovation in a new setting or to new users. This perspective assumes that the innovation itself remains largely unchanged in the process of scaling and that it is crucial to convince users of its utility or value and to create a fitting context. This perspective contributes to our cross-disciplinary framework by highlighting aspects of the local context that are perceived to influence the past and future evolution of the innovation.

The critical perspective assumes that the process of organizational embedding transforms both the context and the innovation to such an extent that the very possibility of scaling a specific innovation needs to be questioned. This perspective does not suggest that scaling up is altogether impossible, but it does emphasize that the innovation itself changes continuously in local scale-up processes as it potentially loses or gains aspects while moving across localities. This perspective contributes to our framework by focusing on the possibility that the innovation itself must adapt during the scale-up process in response to the context to which it is being scaled.

**Table 1:** Summary of the 3 theoretical perspectives on innovation scale-up.

Perspective	Complexities of scale-up	Type of solution
Structural	Structural factors support or hinder successful scale-up of the innovation	Remove structural barriers and strengthen structural facilitators
Ecological	Local context defines whether the innovation can be successfully scaled up	Prepare local contexts for the replication of an innovation
Critical	Innovations cannot be uniformly applied to different contexts and must adapt in response to changes in the context	Rethink scaling up beyond blueprint innovations; acknowledge the mutual transformation of local context and innovation as necessary

### Narrative on the Scale-Up of an eHealth Innovation

#### Case Description

The eHealth technology considered here consists of several measuring devices for physiological variables (eg, weight, blood pressure, and heart rate) and a smartphone app to enable RPM in a Dutch university medical center. Patients perform the measurements at home according to a predetermined schedule, after which the data are automatically sent to the care professionals at the hospital. This eHealth application is meant to encourage healthy behavior in patients by showing them their progress and to allow care professionals to monitor their treatment more closely by sharing continuous health measures. In addition, it greatly reduces the need for outpatient visits. The cardiology department of a Dutch university medical center initially implemented this innovation to monitor a specific group of patients and then attempted to extend it to other cardiology patient populations and other departments. Research framed the innovation as a local success. First, it showed that the health outcomes of patients using the innovation did not differ substantially from those of patients following standard face-to-face care trajectories. Second, it showed that patient experience improved, that patients became more involved in their own care, and that care professionals had better insight into patients’ health (references omitted to preserve anonymity).

#### Data Collection

We performed 14 semistructured interviews (average duration of 60 minutes) with professionals involved in the development, implementation, and scaling of the innovation. All respondents provided informed consent, and interviews were audio recorded and transcribed verbatim. The respondents included 2 researchers who developed the innovation, 4 health care professionals (nurses and doctors), 3 IT professionals, 2 project managers (cardiologists), 1 department manager, and 2 liaisons from a MedTech company. Potential respondents were identified through snowballing, starting with the current project lead, and recruited via email. Recruitment stopped when respondents started referring us to individuals we had already interviewed, thus indicating that we had consulted all the main actors involved in the case. Moreover, while different respondents may have been better or less well acquainted with particular aspects of the innovation’s trajectory, the similarity between their views and arguments around scale-up indicated that we had reached analytical saturation.

Most of the interviews (12 out of 14) were conducted jointly by the first 2 authors (SA and CC) to ensure that the data collection reflected the cross-disciplinary perspectives at the heart of this study’s design. The remaining 2 interviews were conducted by one or the other. One of the interviewers has a background in health care economics/management, and the other has a background in sociology/organization science and studies health care systems. The 2 interviewers jointly developed semistructured topic lists and adapted them to the specific expertise of each respondent. Respondents were asked to reflect on such topics as how they experienced developing or using the innovation, difficulties encountered during its development or implementation, changes in care provision and infrastructure, interaction between stakeholders, and views on the future of the innovation.

#### Ethical Considerations

Given that the data collected involved humans, we applied for ethical approval. Ethical approval for this study was granted by the internal review board of the Erasmus School of Health Policy & Management, Erasmus University Rotterdam (reference: ETH2122-0324). All respondents provided written informed consent prior to the start of the interview and were explicitly told they had the opportunity to withdraw from the study at any time or, without stating reasons, withdraw their permission for the use of their information in the study. Data were deidentified as much as possible as all respondents are only referred to by their function and the specific innovation project is described with the omission of any specific references. No form of compensation was provided to the respondents, as we required only a short amount of their time and the research involved very little to no risk of potential harm for the respondents.

#### Data Analysis

We performed data analysis abductively, going back and forth between the insights from theory and the empirical data, and following guidelines from Braun and Clarke on thematic analysis [38,39]. This paper’s focus on scale-up emerged organically as part of this abductive process. Indeed, we initially aimed to investigate what made the innovation a “successful” eHealth innovation. The respondents questioned this depiction of the innovation, however, as they did not perceive its development as finished. Scale-up emerged inductively from our interview data as a central theme in actors’ attempts to make sense of what they were involved in and spurred the development of our cross-disciplinary theoretical framework. Using this framework, we analyzed the narrative generated by the respondents as they reflected on the past and future evolution of this eHealth technology, including their views on scale-up. Our aim in combining and comparing the different perspectives was to gain a richer understanding of the scale-up of eHealth innovation.

After abductively reassessing the theoretical and empirical data, we identified 6 themes, making use of the qualitative analysis software Atlas.ti 23. The first 3 themes were related to the chronological experiences of the respondents, enveloping the initial development and embedding of the innovation, the gradual adaptation of the innovation within the original setting, and the attempts to replicate the innovation in a new setting. The other 3 themes were related to the issues that respondents experienced from diverging (theoretical) perspectives, either the barriers they experienced from a structural perspective, the complex local factors they experienced from an ecological perspective, or the necessity of mutual adaptation of innovation and context they experienced from a critical perspective. Subsequently, using these themes, we created a narrative piecing together the respondents’ perspectives.

We noted that the respondents’ narrative distinguished between *local* organizational scale-up (instances where the innovation’s scope was expanded to include more dimensions of care practice in the same organization) and *broader* cross-organizational scale-up (replicating the innovation in a new organization). We have therefore structured this section according to these experiences. In Part I, we describe how stakeholders made sense of what happened during the initiation and embedding of the innovation. These early experiences demonstrate the importance of pre-existing circumstances for local scale-up. In Part II, we present respondents’ reflections on the local scale-up and the complexities encountered during this process. Finally, in Part III, we discuss respondents’ current experiences and future ambitions for scaling up their innovation to other organizations.

### Part I: Initiation and Embedding of the Original Innovation

#### Local Aspects Shaping the Facilitating and Complicating Circumstances for Innovation

The innovation project started around 2015 at the cardiology department of a Dutch university medical center. As its initiators reported, the project was driven by the will to innovate the practice of cardiology by moving part of care provision outside the hospital using RPM. However, as one of the respondents described, “it wasn’t like this happened suddenly. [...] The undercurrent was already there” (Project Manager 1). Several respondents referred to this “undercurrent” to describe the facilitating circumstances in the department prior to the project’s initiation. We discuss 3 of these pre-existing circumstances below: the maturity of the department’s IT infrastructure; the highly standardized care pathway for (some) patients; and the facilitating workflow, culture, and resources.

First, in the early 2000s, this department was one of the first in the country to develop and implement an electronic health record (EHR). The in-house EHR gave the department a tailored flexible infrastructure into which RPM devices and data could be integrated. The department also had an internal team of dedicated IT professionals who supported staff in integrating the hardware and software and were available to continuously adapt the EHR structure and its data visualization. As one respondent stated, “that made it easier, let’s say, to add more things to our own electronic patient record. That was in fact a reason [for the innovation’s success]” (Department Manager 1).

Second, the RPM project was built on another project that had introduced a care pathway protocol, thereby restructuring care provision for a patient group. As reported by one of the innovation’s initiators, this standardized care pathway laid the groundwork for RPM: “the idea [of reducing patients’ visits to the clinic] came from the project that had already been running in the department for years” (IT Professional 1). Introducing the care pathway protocol brought up 2 important points for consideration: on the one hand, the numerous physical contact moments between medical professionals and patients, and on the other, the lack of data on these patients between hospital visits. Thus, besides providing clarity on disease progression, and a “very well-defined care track” (Department Manager 1), this project also singled out points for improvement that could be addressed through RPM technologies.

Third, the RPM project’s initiators also highlighted how the practice and culture of cardiology are highly technology-, data-, and innovation-driven. The department prided itself on its early adoption of earlier innovative technologies, such as pacemakers, and for being “used to problems with patients with home monitoring devices.” In turn, this meant that they “already had a very fast technical back office to help patients with their problems” (Department Manager 1). The professional workflow and culture at the department therefore enabled a transition toward technological innovation. In addition, being part of a university medical center allowed the department to invest in several PhD candidates whose research on protocolized care pathways, IT infrastructure, and the innovation itself supported the transformation.

After the innovation’s inception, embedding it required a lot of time, effort, and communication. Many issues emerged, ranging from technical (ie, selecting appropriate monitoring devices and finding ways to integrate data from commercial devices into the EHR) to usability (ie, educating patients and professionals about a new form of care provision and discussing it with them). The department needed to undergo further significant changes to embed the innovation. Below, we discuss examples of changes in professional tasks and roles.

The department’s staff, particularly specialized nurses, had to adapt their tasks and knowledge infrastructure. Crucially, nurses became responsible for interpreting the incoming RPM data and for following up with appropriate actions. This new task required them to respecialize to provide care based on data produced remotely by patients. Specifically, as one of them stated, nurses had to become “aware that [a number] is just a number, […] measured by the patient in the home setting, by a device of which I'm not sure how old or how reliable it is” (Nurse 1). This required them to view measurements as not always trustworthy (as numbers that offered guidance but could not be taken at face value). This new orientation stemmed from firsthand experiences on the job and from sharing these experiences with their colleagues. Furthermore, the department created a new role: “eHealth consultants,” tasked with distributing the innovation to patients, instructing them in its use, and addressing technical questions. By hiring eHealth consultants, the department acknowledged the work needed to guide patients in their use of the innovation and formally integrated tasks previously conducted informally by PhDs into the organizational structure. These organizational changes resonate with the ecological perspective in our theoretical framework.

#### Complexity Also Lies in a Process of Mutual Adaptation of Innovation and Context

Embedding the innovation in the department also involved adapting the innovation itself, reflecting the critical perspective articulated above. For example, the IT department, in collaboration with local nurses, developed a new dashboard in the EHR to visualize incoming data. They also installed secured software to enable e-consultations with patients and developed an app to give patients personalized instructions. The hospital’s physical infrastructure was also adapted. Specifically, the department transformed a central space in the hospital into an office where patients could meet the eHealth consultants and discuss their questions. As explained by a cardiologist:

At this point there also came a different kind of department that had a focus on the innovation only, so they make sure that the patient gets the devices, they make sure that everything is electronically connected the right way, that the data are coming in, they always call the patients within 2-3 weeks to make sure that everything is going well, that the data are coming in. So I think that was crucial.

Doctor 1

#### Concurrently, Structural Barriers Need to be Addressed

While the respondents were busy preparing the local context for the innovation and at the same time adapting the innovation itself, barriers of a structural nature were addressed but not yet solved permanently (ie, negotiating issues of safety, financing, and medical device regulation). For example, all the infrastructural changes were financed through the departmental budget and temporary research grants because there was no regular reimbursement available for these types of care provisions. According to the respondents, this was possible only because the cardiology department had access to more financial resources than other departments in this and other hospitals. Moreover, the department was responsible for the care pathway of the patient target group from beginning to end, giving them the autonomy necessary to transform care provision. An external project manager explained:

Because it’s only their department, that makes them really quick in making decisions and going forward.

Project Manager 2

Analyzing the innovation’s organizational embedding highlights the necessity of addressing structural financial and regulatory barriers early on and the need for a flexible and continuously developing local infrastructure. In addition, the innovation itself changed in response to the requirements of the local setting. With regard to embedding, the respondents’ narratives resonate with the structural, ecological, and critical perspectives.

### Part II: Gradual Adaptation of the Innovation Within the Original Setting

We now turn to an analysis of the innovation’s evolution from its initiation (2015) to the period of data collection (2022). This period saw a local scale-up in terms of (1) the patient population covered by the innovation, (2) the technical aspects of the innovation, and (3) the number of departments using the innovation. Each of these expansions of coverage faced several complexities.

#### Aspects in the Local Setting That Complicate Gradual Scale-Up

Some of these complexities relate to the ecological perspective. To begin with, local scale-up focused on replicating the innovation within the cardiology department to cover new patient groups. This had major consequences for the care professionals involved (again, nurses in particular). Patient numbers and data collection requirements increased, placing a significant burden on the department’s care professionals, who perceived the amount of data generated by up to 400 patients daily as overwhelming. Moreover, scaling up to other patient populations increased uncertainty in nurses’ daily tasks, leaving them unable to plan and putting them under more stress. One respondent explained:

If you have patients who are continuously doing these measurements at times that they find suitable, they contact you at unpredictable times with questions that can be emotional, that can be technical, they can be completely fine, but they can also be extremely ill, and you must adjust your actions as a medical professional accordingly. That type of unpredictability, when you don’t know at the beginning of the day where it’s going to end, that just introduces some stress.

Doctor 2

Several nurses decided to “evaluate critically how often they check those measurements, because it is such a huge investment of time and such a burden” (Nurse 2). Nurses started looking critically at the real benefits of the deluge of data they received and began to wonder whether *“*it could potentially be better to place the responsibility with the patients” (Nurse 2). As a result, patients were increasingly instructed to keep an eye on their own data and explicitly made responsible for contacting the hospital if they believed something was wrong.

#### Gradual Scale-Up Also Requires Continuous Adaptation of the Innovation Itself

Complexities described by the respondents also resonate with the critical perspective, since the innovation had to be adapted based on the needs and possibilities of the local context. For instance, the project leads continuously added hardware (ie, new measuring devices) to the innovation to provide more extensive data. One of them explained:

What we’re trying to do is check whether there are more non-invasive devices that you can combine to make sure that you can see that the patient is developing heart failure again as soon as possible. So that’s why we’re continuously monitoring and checking, okay, what could we add that could possibly help us? Because now we only have the step counter and the weight scale and the blood pressure monitor, but maybe, just maybe, it will help us if we can look at the sleep monitor as well. Because there will be some data that we can combine.

Doctor 1

Since new hardware and datapoints would further exacerbate the data issues experienced by nurses, the IT department, in collaboration with nurses, started developing an artificial intelligence (AI) model aimed at analyzing the growing amount of patient data and flagging those patients in need of nurses’ attention. Although the model had not been implemented yet, many of the respondents agreed that AI was necessary for the innovation to be workable in practice, even more so given the scaling-up ambitions.

All these add-ons made it difficult to draw clear boundaries around the innovation. In addition, the innovation increasingly became an unstable object for local stakeholders, which in turn complicated further scale-up plans. One doctor stated:

The project has not been finalized yet. […] So, I would say, in order for it to be implemented in other hospitals, you need to come to at least a sort of 1.0 solution in which you have a proper and clear description of the product service design, [but] we are still designing it as we go along.

Doctor 2

Interestingly, despite not considering the innovation as a finished product, local professionals still attempted to scale it to other departments, especially following strategic investments from the hospital. As the context of scale-up started to move away from the original department, the stakeholder strategies that had functioned in the local context began to fall short. Stakeholders attempted to generate a process of mutual adaptation between local context and innovation, replicating the continuous changes that the “original” innovation underwent at the cardiology department. Many respondents acknowledged, however, that it was inherently much more difficult to scale the innovation to other hospital departments.

#### Structural Barriers Impede the Gradual Scale-Up of the Innovation

The factors impeding the scaling of the innovation within the hospital also resonate with the structural perspective, for example, the limitations of the external IT infrastructure adopted in other departments, their tight budgets, and the lack of eHealth consultants and dedicated IT staff there. Respondents reported being able to work around some of these barriers, for instance, by drawing on temporary grants and budgetary slack to compensate for the lack of structural reimbursement and by postponing discussions around regulatory safety and liability. One respondent explained:

Everything we have done so far was a little bit in this department, a little bit in that department. We had different ways of presenting the data [from patients], we had different IT infrastructural routes; […] the hospital picked five different departments and said, “every department gets some money for fifty patients per [RPM innovation], they get some money from a grant”. […] For now, the departments, they arrange the technical explanations to the patients themselves. But, if we get new [versions of the innovation addressing different conditions], you know – the workload is already quite high with the nurse practitioners… […] In an ideal world, you would like to have an overall eHealth department that can do the support of all the [innovations in different departments]. I think that would be necessary if we were really scaling up the [innovation] within the [hospital].

Project Manager 2

To summarize, local scaling of the innovation within the organization had its challenges, with respondents recounting organizational complexity, mutual adaptation of context and innovation, and structural barriers. Yet despite these complexities, we can conclude that local scale-up successfully extended the innovation’s coverage to include more patients, more aspects of the care pathway, and more departments.

### Part III: Attempts to Replicate the Innovation in a New Setting

At the time of our interviews, the respondents considered their innovation as one that was still evolving with the gradual addition of new software, hardware, and patients. They also envisaged further replications of the innovation in departments and hospitals nationwide. Generally, they viewed broader scale-up attempts as inevitable. Although many respondents mentioned this as a concrete possibility in the (near) future, they were well aware of the associated complexities.

#### Aspects of Local Complexity Remain Important Issues for Replication Attempts Across Settings

To begin with, respondents recognized the organizational complexity, arguing that local people and infrastructure cannot be ignored when trying to implement the innovation in another setting, a point clearly reminiscent of the ecological perspective in our framework. In terms of local people, for example, they referred to the attitude of health care professionals toward innovation and to the different ways in which patients interacted with it. One significant challenge, in their view, lay in convincing medical professionals and local managers to support and adopt RPM, a relatively new type of care provision, because “at this moment […] it’s very difficult to prove that it’s better than what we used to do” (Doctor 2). One respondent reflected on the attempts to scale up more widely:

If you start this, you start with the small groups who are believers, curious people. So, they are motivated to do it. The difficult part comes after that, when you have to scale up, introduce more people to this way of care. And then you find people who say “oh this is extra, I have to look at all the data, I don’t have time for that!” We have to explain to them that this is part of our journey.

Project Manager 1

In terms of local infrastructure, respondents expected issues to arise around the local IT systems and the funds available to invest in all the necessary adaptations:

The other thing is, let’s say in terms of the cost structure, if we want to bring this to other hospitals, you also have to think about a lot of logistics. So, the innovation requires centers. [Hospital] has created an office, where you can go and get your stuff as a patient. All that is taken care of. It’s not so easy to replicate that in other places [...] with minimal costs. And there are also hidden costs. For example, let’s say, the personnel costs are not calculated by [hospital].

Liaison 1

The farther away the innovation moves from the epicenter of its origin, the more difficult it seemingly becomes to get the innovation embedded in the organizational setting: 

The difficulty we have is that the infrastructure of the innovation is now really incorporated in the [hospital] infrastructure. So, if we want to expand to other hospitals, we need to get to a plug-and-play solution, that you get the app with a dashboard that you can connect into as a hospital. To connect them with the [hospital] infrastructure, that is also a step that needs to be made.

Project Manager 2

#### Resolving Structural Barriers Presents One Solution to Stagnating Scale-Up

The respondents emphasized the necessity of a plug-and-play innovation to resolve issues on a broader level, and this is a solution that reflects the structural perspective discussed earlier in this paper. Respondents struggled to articulate viable strategies for overcoming these systemic barriers. This has been illustrated by the following quote:

In the short term the business plan behind mobile health technology is bankruptcy. It just means bankruptcy for a classical hospital, so to say. So, there are a lot of hurdles that must either be taken or ignored in order to make this a success.

Doctor 2

Unresolved structural barriers mentioned included health care providers not being reimbursed, or not enough, for avoiding unnecessary visits to the hospital; health insurers having to choose from among a growing number of potential eHealth innovations; and a lack of resources for insurers with the largest market share in a region, who would be expected to take the lead in investing in innovations. One respondent summarized:

It has nothing to do with the technology, it’s purelya cost versus revenue issue. […] that’s what makes it difficult to bring this to other organizations.

Liaison 1

In addition to financial issues, participants recognized structural barriers to broader scale-up of eHealth innovations in regulatory and quality constraints, in the fragmentation of IT systems used throughout the country, in hospitals’ perceived risk aversion when it comes to investing in innovations, in the absence of suitable hospital infrastructure and new professional roles supporting eHealth care provision, and in the environmental unsustainability of data storage and single-use medical devices. Nevertheless, respondents did emphasize that they expected these issues to be resolved over time:

Like I said, there is hope, we just need to accept that the way we organize our health insurance system is not going to help us implement digital solutions. There is enough awareness, so I guess at some point in time we will come up with a solution, but it’s going to take time.

Doctor 2

Moreover, respondents considered several strategies for dealing with the structural complexities:

We are working on the general issues, for instance liability, ethics, data ownership. We organize meetings with all the (national) institutes that are responsible, we convene. Because everybody needs the same answers. They have the same questions at least.

Project Manager 1

In addition, they recounted that collaborations with private companies had been considered to support broader scale-up, often framed as “commercialization” (Researcher 2). In this scenario, selling the innovation to an independent organization would allow them to outsource legal liability and the development of an independent IT infrastructure. The strategies respondents described to address structural barriers in reimbursement and regulatory systems, however, assumed that the innovation was a finished product. For the insurer to provide a reimbursement code, authorities to provide certification, or a private company to sell the innovation, there needed to be agreement on a stable and finalized innovation.

#### Acknowledging the Malleability of the Innovation Presents Another Solution to Stagnating Scale-Up

While some respondents described the innovation as always boiling down to “the same object” (IT Professional 2) regardless of setting, others held the view that it could not simply be considered “a thing” that was reproducible across settings. Consistent with the critical perspective identified above, these respondents claimed that every instantiation of the innovation would in fact amount to another entity altogether, because the innovation had to shed some features and acquire new ones in order to work locally. This narrative problematized the depiction of the innovation as a stable technological object:

[Although] there is one message and one goal, […] the [innovation] has been expanded from that original one. [...] See, you see an [innovation]. But it is the whole idea behind it, it’s the concept. It's not a technology, it's a concept. That can be difficult to explain.

Project Manager 1

These “critical” respondents questioned what constituted the core of the innovation. Was it a form of care provision at home with the involvement of remote technology? A preventative intervention to keep patients out of the hospital? Or nothing more than a concept, an idea about the values of contemporary health care? These reflections (ie, which of the core features of the innovation had to be replicated for the replication to count as scale-up) have important implications for how we appreciate the complexities of broader scale-up. One respondent commented:

[N]obody cares about the devices. How are you selling something that is a way of working right? How are you selling change management? [...] Maybe the [innovation] is more like a consultancy service that you buy as a hospital. [Liaison 1]

In summary, in trying to make sense of the complexities of broader scale-up, our respondents were caught up in a paradox. On the one hand, they considered the innovation a clearly demarcated product subject to financial and regulatory issues. On the other hand, they also acknowledged that it needed to be malleable to deal with the complexities of transitioning to another context. In our discussion, we reflect on this tension and draw lessons from it for both theory and practice.

## Key Insights and Discussion

### Overview

To dissect the complexities involved in scaling up eHealth innovations, we have combined different theoretical perspectives to make sense of the narrative constructed by stakeholders involved in the scale-up of an innovative eHealth technology. Based on insights from different fields of literature, we have (in brief) identified a structural perspective focusing on systemic barriers and facilitators, an ecological perspective focusing on local organizational complexity, and a critical perspective focusing on mutual adaptation of context and innovation. The 3 perspectives provide complementary explanations for the complexities perceived in the scale-up of eHealth innovation. The structural perspective, for example, aligned with respondents’ observations regarding flawed reimbursement systems and fragmented IT infrastructure within and between Dutch hospitals. The ecological perspective resonated with respondents’ reflections on the importance of convincing medical professionals of the innovation’s value, as well as on the reconfiguration of nurses’ tasks and the establishment of a central eHealth office to improve workflow. The critical perspective was consistent with respondents acknowledging the ongoing evolution and adaptation of the innovation, for example, with the addition of novel monitoring devices and AI-powered software.

Our analysis thus shows the importance of adopting a cross-disciplinary perspective when examining eHealth scale-up and its associated complexities. While the structural perspective tends to overlook the flexibility and malleability necessary for scaling an innovation to another organizational context, the ecological and critical perspectives fail to connect local experiences with the stability perceived to be required of the innovation to reach upscaling at broader levels. Below, we elaborate on 2 distinct insights that emerged from this combination of perspectives and discuss their implications for researchers, practitioners, and policy makers.

### Differing Strategies Used for Scaling Up eHealth at Different Levels of Scale

While the respondents’ narratives bore traces of each of the 3 perspectives, the strategies they used to deal with scale-related complexities differed considerably depending on the level (ie, local versus interorganizational). When discussing *local scale-up*, the respondents stated that they worked on changing the context, adapting practices and infrastructure to embed the innovation, and reconfiguring the innovation itself. In this sense, the flexibility of the organization and innovation was crucial. Although respondents mentioned several structural barriers as having affected the progress of the innovation (eg, the lack of reimbursement agreements with health insurers), they did not resolve them formally but rather worked around them informally.

In contrast, when reflecting on the plans for *broader scale-up*, respondents questioned the strategy of informality and flexibility. When it came to scaling the innovation to other organizations, respondents emphasized the need to find formal solutions to systemic barriers, such as national reimbursement arrangements, an integrated IT network, and national regulatory and liability agreements. Moreover, to realize this, the innovation itself had to cease being malleable and become a stable “product.” The view of innovation as a formalized stable entity stems from the structural perspective, which assumes that innovation is a thing embedded in formal structures. In contrast, the critical perspective emphasizes how scaling an innovation to another context entails changing what the innovation *is*. The idea that the innovation never becomes a stable entity amenable to replication does not align with the perceived necessity of turning it into a clearly demarcated product. This tension has critical implications for the solutions respondents envisage for dealing with complexities at a broader level of scale-up: proposing concrete solutions to overcome systemic barriers assumes that the innovation can achieve a state of closure, and that is not what the respondents experienced.

To summarize, our analysis revealed a tension between 2 different conceptions of eHealth innovation: as something that is malleable *and* entangled in an organization, and as a stable product that can be replicated across contexts without changing. This tension results in an impasse in developing strategies to overcome the complexities involved in broader scale-up, with respondents being incentivized to keep the innovation malleable and fixed at the same time.

### Importance of Path Dependencies Shaping Chances for Scale-Up

In addition to the important role of the level of scale, we identified another aspect that is often overlooked in understanding the complexities of scaling up eHealth innovation. Prior research has often advocated establishing the infrastructure needed for local scale-up without *acknowledging the influence of what already exists*. Although the ecological perspective emphasizes the need to adapt the local setting, what is missing from this view is the path dependencies stemming from previous decisions. Defined by Mahoney [40] as “those historical sequences in which contingent events set into motion institutional patterns or even chains that have deterministic properties,” path dependencies refer to how current events depend at least in part on a chain of prior events. When translating this to innovation scale-up, it becomes clear that success is predicated not only on present and future actions but also on how past choices have shaped organizational structures. It is unlikely that all local circumstances can be adapted to accommodate an existing innovation; some are likely to complicate eHealth scaling. An example can be found in the discussion surrounding the pre-existing IT infrastructure of the cardiology department where the RPM innovation originated. The presence of an in-house EHR allowed for a tailored flexible infrastructure into which the innovation’s devices and data could be integrated, something that would have been much more difficult in a commercial platform. However, the use of such platforms is the prevailing reality in most hospitals in the Netherlands.

In summary, pre-existing local circumstances can significantly shape eHealth innovations’ initiation and scale-up, and the associated path dependencies should be taken into account when attempting eHealth scale-up both locally and more broadly.

### Recommendations for Practice and Policy

This article offers several lessons for practice and policy. First, the challenges faced by actors at the local and broader levels are likely to differ greatly. Actors at the local level are likely to face complexities related to the continuous adaptation of both the organizational infrastructure and the innovation itself. Even so, there is little sense of urgency at this level about addressing structural barriers formally and systematically. In contrast, actors at the broader level need to deal with the tension between the limitations imposed by structural systems and the requirement of local flexibility. To do this, decision makers must, first and foremost, acknowledge this tension and the confusion that is likely to result regarding the strategies that actors pursue in their attempts to scale up broadly.

The second lesson, related to the previous point, is that flexibility should be incorporated into system-wide structures that govern eHealth innovation (including IT infrastructure, regulations, and reimbursement mechanisms). Specifically, this would mean creating opportunities for local adaptation work, facilitating ingenuity in local contexts. While fostering such “facilitating space” will not remove all complexity, it will help actors deal with complexity by giving them a certain amount of leeway to tailor and adapt to the requirements of specific local contexts. This complexity includes the path dependencies imposed by pre-existing circumstances that define the opportunities for scaling up specific innovations.

That is why actors should be aware that, when adopting a scaled-up innovation, they will likely need to adapt the innovation itself as well as the local context. At the policy level, such awareness should be made part of reimbursement and regulatory structures. For decision makers, this means not expecting a “plug-and-play” model of scale-up where innovation is simply replicated; rather, they must set aside the time and financial and human resources necessary for adaptation at the local level.

### Recommendations for Research

Our analysis leads us to make 3 recommendations for future research. First, despite discussing scale-up at length, existing literature lacks a conceptual vocabulary for studying the concept of scale itself, including its implications for the complexities stakeholders face and the strategies they use. There is almost no research addressing the phenomenon of scale in innovation scale-up, the notable exception being a study of strategies addressing scale-up complexities at different levels of scale in the energy sector [41]. Future research on health care innovation scale-up should thus acknowledge the concept of the scale itself (ie, the level at which scale-up is attempted) as a crucial factor in determining actors’ strategies.

Second, research should focus on whether and how actors involved in different types of innovations may experience complexities at different levels of scale. The RPM innovation studied in this paper is only one of many types of eHealth innovations, each with specific characteristics that may affect the process of scaling. Others could, for example, have more stable or more malleable aspects and come up against more or fewer unresolved systemic barriers. More research is needed to further our understanding of these complexities and identify a broader set of action repertoires to deal with them.

Third, this article adopted a cross-disciplinary perspective, applying a generative heuristic framework to highlight the various complexities stakeholders encounter in the scale-up of eHealth innovations through a high-level discussion. Consequently, our analysis leaves considerable scope for research on more specific aspects of these complexities. One potentially relevant direction for research could be the interaction of humans with technologies, the novel challenges emerging from this interaction in care processes [42], and the ways in which this interaction and the related challenges may differ based on the scale attempted.

### Limitations Related to the Case Study

There are several limitations associated with our case study. First, for reasons of unavailability and privacy protection, we were unable to include the perspectives of all stakeholders currently involved in the RPM innovation project. Specifically, we did not include the perspectives of eHealth consultants, patients, and stakeholders in other departments outside of the original cardiology setting. Stakeholders in departments attempting to scale the innovation could have provided additional insights into the complexities of scale-up. We were also unable to speak to the nurses directly involved in the original version of the innovation owing to the work pressure they were experiencing. We did, however, talk to their colleague nurses, who witnessed how the workload associated with the innovation proved demotivating for several nurses.

Second, several biases could have emerged from the interview-based nature of the case study. Specifically, both the retrospective (recall bias, potentially misremembering events in the past) and prospective (declinism, the tendency to perceive the future more negatively than the past) nature of the respondents’ reflections might have shaped our findings. We cannot preclude the possibility that we missed relevant aspects, as we were unable to observe the innovation’s development as it happened. However, the interviews did allow us to piece together a longitudinal narrative spanning a much longer time period than any direct observations would have allowed. Moreover, we did reach analytical saturation based on our interviews, insofar as respondents presented similar views on scale-up across interviews, strengthening our confidence in the validity of our conclusions.

### Conclusion

This article addressed the question of why scaling up innovative eHealth technologies is so challenging in practice. For this purpose, we brought together 3 theoretical perspectives on the complexities of innovation scale-up from different fields of literature. We used these perspectives to make sense of the narrative produced by stakeholders involved in the scale-up of an RPM-based eHealth technology, which was presented as a local success but came up against challenges in broader scale-up attempts. Each of these perspectives highlights different but equally pertinent aspects of scale-up complexities and strategies for addressing them. Two key insights emerged from this cross-disciplinary analysis. First, we found that the level at which scale-up is pursued plays an important, yet so far neglected, role. Contextual complexities were overcome at the local level and systemic barriers were informally worked around. By contrast, at a broader level, tension emerged between the need for stability on the one hand and malleability on the other, leading to an impasse in the scale-up of the eHealth innovation. Second, our study has emphasized the role of path dependencies, namely in terms of pre-existing organizational structures and technological infrastructure, in facilitating and constraining scale-up processes. The path dependencies in local contexts play an important role in shaping the complexities that actors face.

Researchers, policy makers, and stakeholder practitioners need to acknowledge and account for the crucial roles that the level of scale and path dependencies play in shaping the complexities involved in scaling up eHealth innovations. Such projects might enjoy greater success by rethinking structural systems to allow for malleability in the innovation, giving it the necessary leeway to align with the requirements of specific local contexts.

## Abbreviations

AI: artificial intelligence
EHR: electronic health record
RPM: remote patient monitoring
